# Genetic connectivity of lionfish (*Pterois volitans*) in marine protected areas of the Gulf of Mexico and Caribbean Sea

**DOI:** 10.1002/ece3.5829

**Published:** 2020-04-16

**Authors:** Irán A. Guzmán‐Méndez, Renata Rivera‐Madrid, Serge Planes, Emilie Boissin, Aldo Cróquer, Esteban Agudo-Adriani, Carlos González‐Gándara, Horacio Perez‐España, Ana Giro‐Petersen, Jenny Luque, María del C. García‐Rivas, Margarita Aguilar‐Espinosa, Jimmy Arguelles Jiménez, Jesus E. Arias‐González

**Affiliations:** ^1^ Laboratorio de Ecología de Ecosistemas de Arrecifes Coralinos Departamento de Recursos del Mar Centro de Investigación y de Estudios Avanzados del I.P.N.‐ Unidad Mérida Mérida México; ^2^ Department of Biological Sciences Marquette University Milwaukee WI USA; ^3^ Unidad de Bioquímica Molecular de Plantas Centro de Investigación Científica de Yucatán Mérida México; ^4^ PSL Research University: EPHE‐UPVD‐CNRS USR 3278 CRIOBE Université de Perpignan Perpignan Cedex France; ^5^ Laboratoire d'Excellence « CORAIL » Perpignan Cedex France; ^6^ Departamento de Estudios Ambientales Universidad Simón Bolívar Caracas Venezuela; ^7^ Laboratorio de Arrecifes Coralinos. Carrera de Biología Universidad Veracruzana Tuxpan México; ^8^ Instituto de Ciencias Marinas y Pesquerías Universidad Veracruzana Boca del Río México; ^9^ Healthy Reefs for Healthy People Initiative Ciudad de Guatemala Guatemala; ^10^ Bay Islands Association Utila Honduras Utila Honduras; ^11^ Comisión Nacional de Áreas Naturales Protegidas Parque Nacional Arrecifes de Puerto Morelos Puerto Morelos México

**Keywords:** Caribbean Sea, founder event, genetic structure, invasive species, lionfish, marine protected areas, microsatellites

## Abstract

Lionfish (*Pterois volitans*) have rapidly invaded the tropical Atlantic and spread across the wider Caribbean in a relatively short period of time. Because of its high invasion capacity, we used it as a model to identify the connectivity among nine marine protected areas (MPAs) situated in four countries in the Gulf of Mexico and the Caribbean Sea. This study provides evidence of local genetic differentiation of *P. volitans* in the Gulf of Mexico and the Caribbean Sea. A total of 475 lionfish samples were characterized with 12 microsatellites, with 6–20 alleles per locus. Departures from Hardy–Weinberg equilibrium (HWE) were found in 10 of the 12 loci, all caused by heterozygous excess. Moderate genetic differentiation was observed between Chiriviche, Venezuela and Xcalak, México localities (*F*_ST_ = 0.012), and between the Los Roques and the Veracruz (*F*_ST_ = 0.074) sites. STRUCTURE analysis found that four genetic entities best fit our data. A unique genetic group in the Gulf of Mexico may imply that the lionfish invasion unfolded both in a counterclockwise manner in the Gulf of Mexico. In spite of the notable dispersion of *P. volitans*, our results show some genetic structure, as do other noninvasive Caribbean fish species, suggesting that the connectivity in some MPAs analyzed in the Caribbean is limited and caused by only a few source individuals with subsequent genetic drift leading to local genetic differentiation. This indicates that *P. volitans* dispersion could be caused by mesoscale phenomena, which produce stochastic connectivity pulses. Due to the isolation of some MPAs from others, these findings may hold a promise for local short‐term control of by means of intensive fishing, even in MPAs, and may have regional long‐term effects.

## Graphical abstract

Our findings demonstrate that there is genetic differentiation at local and regional scales.We suggest that the colonization of each geographic area has been temporary pulses, probably caused by mesoscale phenomena, and intensified with the presence of self‐recruitment. The genetic diversity of *Pterois volitans* in the Gulf and the Caribbean showed that the number of organisms that originated the invasion was much higher than the one mentioned in the literature.

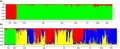

## 1 INTRODUCTION

The lionfish species complex is composed of two congeneric species (*Pterois volitans* and *Pterois miles*) that were initially introduced on the coast of Florida. The first sighting occurred in 1985 (Schofield, 2009). By 1992, the population had increased and dispersed rapidly to the north, following the coast of the United States toward New York, and later progressing south to the Bahamas (Betancur‐R et al., 2011). Currently, lionfish have spread throughout the Caribbean and have recently reached Brazil (Ferreira et al., 2015). Several features have made lionfish extremely good invaders. First, females can release 10,000–40,000 eggs at a spawning event (Morris & Akins, 2009) and show asynchronous releases (Morris, Sullivan, & Govoni, 2011; Murua & Saborido‐Rey, 2003). Second, lionfish are opportunistic feeders, and feed widely on fish and crustaceans, with a list of more than 70 different species included in their diet (Valdez‐Moreno, Quintal‐Lizama, Gómez‐Lozano, & García‐Rivas, 2012). Third, lionfish possess venomous spines that prevent most native predators from eating them (Morris & Akins, 2009). Finally, lionfish can occupy a range of habitats from shallow waters to waters 300 m deep (Albins & Hixon, 2013) and tolerate a broad range of salinity (Jud, Nichols, & Layman, 2014). Little is known about the behavior of *P. volitans/miles* larvae; it is only known that they are dispersed by ocean currents and they have then 35–40 days of the pelagic stage (Morris, Akins, Barse, Cerino, & Freshwater, 2008; Freshwater et al., 2009). No real coordinated controls are in place for the larger region, but local control strategies involve intensive fishing of lionfish included the Marine Protected Areas (MPAs).

Marine protected areas are tools used for the administration and management of marine resources (Pomeroy, Watson, Parks, & Cid, 2005), and their success depends on connectivity among other MPAs to promote population persistence in marine ecosystems (Planes, Jones, & Thorrold, 2009). Connectivity has always been a key point in the success of MPAs strategies. The connectivity process allows transportation of organisms between populations, contributing to increased genetic diversity and colonization from distant populations (Palumbi, 2003). Connectivity occurs through larval dispersal and adult migration, the former being the primary mechanism of connectivity between reefs (Sale et al., 2010). Currently, in ecology, different definitions are used for connectivity, depending on the context, and the type of evaluation method (Lowe & Allendorf, 2010). Genetic connectivity is the amount of gene flow occurring among populations over a timescale of several generations. It determines the extent of genetic differences among populations (Sale et al., 2010). Even when the connectivity assessment is specific and unique to one species, biological models are frequently used to predict the behavior of other species with similar characteristics. Several connectivity studies have been carried out in the Caribbean, using predictive models (Abesamis, Stockwell, Bernardo, Villanoy, & Russ, 2016; Cowen, Paris, & Srinivasan, 2006) to direct genetic studies of reef species (Bakker et al., 2016; Rippe et al., 2017; Villegas‐Sánchez, Pérez‐España, Rivera‐Madrid, Salas‐Monreal, & Arias‐González, 2014; Villegas‐Sánchez, Rivera‐Madrid, & Arias‐González, 2010). However, to date, regional connectivity among MPAs in the Caribbean and the Gulf of Mexico along the Mexican coasts has not been estimated through direct methods. Studies of lionfish in the Caribbean have explored the use of molecular technologies for the analysis of the populations of *P. volitans/miles* from different approaches (Betancur‐R et al., 2011; Bors, Herrera, Morris, & Shank, 2019; Butterfield et al., 2015; Johnson, Bird, Johnston, Fogg, & Hogan, 2016; Pérez‐Portela et al., 2018; Toledo‐Hernández et al., 2014; Freshwater et al., 2009). However, the current study is the first to use microsatellites. The main objective of this study was to explore the level of genetic connectivity among *P. volitans* populations in Marine Protected Areas in the Gulf of Mexico and Caribbean Sea, using specific microsatellite markers for *P. volitans/miles* (Schultz, Fitzpatrick, Wilson Freshwater, & Morris, 2013). The rapid dispersion of this specie and environmental geographical barriers could influence locally differentiated populations, making it possible to evaluate connectivity matrices. Investigating this hypothesis may help us understand the dispersal behavior of this invasive species on a local scale and help develop guidelines for effective local and regional management.

## 2 METHODS

### 2.1 Collection of biological material

**Figure 1:**
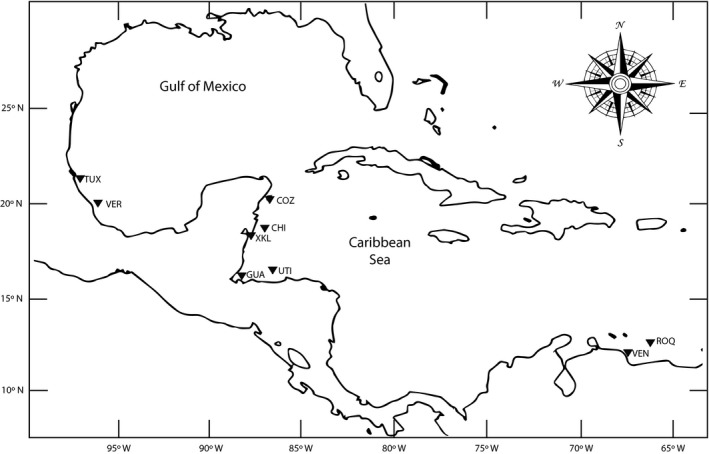
Map of sampled Marine Protected Areas **(**MPAs) from north to south and from left to right: Tuxpan (TUX), Veracruz (VER), Cozumel (COZ), Chinchorro (CHI), Xcalak (XKL), Guatemala (GUA), Utila (UTI), Chiriviche (VEN), and Los Roques (ROQ)

All individuals were collected within MPAs by scuba divers using Hawaiian harpoons. A total of 475 *Pterois* were sampled between May 2013 and January 2015 from nine MPAs. These included five localities in Mexico: Tuxpan (35), Veracruz (13), Cozumel (76), Chinchorro (77), and Xcalak (89); one locality in Guatemala: Punta Manabique (44); one locality in Honduras: Utila, (54); and two localities in Venezuela: Los Roques (32) and Chiriviche (55) (Figure 1). As part of the *Pterois* species local control strategies, personnel in charge of each MPA were involved in collecting the individuals. A fragment of the caudal peduncle muscle was extracted from each sample, preserved in 70% alcohol and stored at 4°C until DNA extraction and analysis were performed at the laboratory. The current study concentrates on the most abundant species, *P. volitans*.

### 2.2 DNA extraction and identification of *P. volitans*

Genomic DNA was extracted from all samples using the DNeasy Blood and Tissue Kit (Qiagen) following manufacturer's instructions. To ensure that every individual had been correctly identified as *P. volitans*, all samples were tested using the methodology described by Guzmán‐Méndez, Rivera‐Madrid, Díaz‐Jaimes, Aguilar‐Espinosa, and Arias‐González (2017).

### 2.3 Microsatellites

We examined 12 polymorphic microsatellite loci for *P. volitans* and *P. miles* published by Schultz et al. (2013). Nineteen nucleotides (5′‐CACGACGTTGTAAAA CGAC‐3′) were added to the 5′‐end of every primer to incorporate primer M13 marked with 5′‐IRD800 or 5‐IRD700 fluorescence (LI‐COR). PCR reactions with a final volume of 15 μl were performed as follows: 0.3 units Taq Platinium (Invitrogen 10966‐030); 0.12 μl dNTP Mix (Invitrogen 18427‐013) at 100 mM; 0.9 μl MgCl2 at 50 mM; 0.5 μl of each primer at 1 pmol/μl (including the M13 marked primer); 1.5 μl DNA at a concentration of 20 ng/μl; and 1.5 μl 10× PCR Rnx Buffer without MgCl2 (Invitrogen). Amplifying conditions were as follows: 4 min at 94°C; 25 cycles of 15 s at 94°C, 15 s at 62°C, and 30 s at 72°C, as described by Schultz et al. (2013). We added eight cycles of 15 s at 94°C, 15 s at 53°C, and 30 s at 72°C with a final extension at 72°C for 5 min to incorporate the M13 primer. PCR reactions were analyzed using LI‐COR Model 4300 DNA and microsatellite sizes were determined with Saga 3.1.2 software.

### 2.4 Data analysis

Each microsatellite was checked with Micro‐Checker (Van Oosterhout, Hutchinson, Wills, & Shipley, 2004) to identify disequilibrium and potential false positives, looking out for important changes in the size of the bands, predominant amplification of small alleles, absence of gel bands or any other typographic errors.

We calculated the following parameters: (a) average number of private alleles per locus; (b) allelic richness, both per locus and population; and (c) Nei's genetic diversity. Based on heterozygosity of allelic frequencies we estimated: (a) Nei's fixation index (*F*_IS_), (b) average expected total heterozygosity, (c) average expected heterozygosity by population, and (d) coefficient of gene differentiation (*G*_ST_) for each locus. Finally, frequency of null alleles (Na) for each population and locus was estimated. Genetic structure analysis started with computing a principal coordinate analysis (PCA) on genetic frequencies to plot the samples in a reduced dimensional map and identify the most divergent locations (i.e., MPAs). We then computed a hierarchical analysis of molecular variance (AMOVA) carried out with 999 permutations to corroborate the statistical baseline of the main divergence observed in the PCA. In addition, we calculated the *F*_ST_ values between all sites also to validate the structure and we made a sequential Bonferroni adjustment for multiple tests (Sokal & Rohlf, 1995). Additionally, we calculate a pairwise Hedrick's standardized $F_{\mathrm{ST}}^{\prime }$ index, which is a standardized measure of population differentiation. This distance is allowing comparison between studies with different types of markers and different effective population sizes (Hedrick, 2005; Meirmans, 2006; Meirmans & Hedrick, 2011). All calculations were made with GenAlEx 6.5 (Peakall & Smouse, 2006) and with Genetix (Belkhir, Borsa, Chikhi, Raufaste, & Bonhomme, 2001).

Lastly, we investigated the genetic structure using a Bayesian approach considering all individuals within the same genetic entity. STRUCTURE 2.3.4 was used to optimize the variance in each genetic population. The analysis was conducted with 100,000 generations for *burnin* and 500,000 recorded generations for each *K*, repeated, for *K* = 1 to *K* = 9, 10 times for each *K*. Then, we used sampling locations as prior (model LOC‐PRIOR, Hubisz, Falush, Stephens, & Pritchard, 2009) for three regions as locality priorities. The number of the most likely clusters was determined using the ∆*K* approach (Evanno, Regnaut, & Goudet, 2005), using Structure Harvester (Earl, 2012).

## 3 RESULTS

A total of 143 alleles were found in the 12 microsatellite loci at nine sites of 475 individuals. Observed allele size range (bp) and number of alleles (Na) in each locus were slightly wider than published by Schultz et al. (2013), with a range of 20 to 6 alleles per locus for an average of 468 amplified samples (Table 1). Departures from Hardy–Weinberg equilibrium (HWE) were found in 10 of the 12 loci (Table 1). Pvm11 locus showed departures at eight of the nine sites; Pvm12, Pvm31, Pvm32, and Pvm46 had departures at seven sites; Pvm15 and Pvm42 had departures at six sites; Pvm4 had departures at five sites; and Pvm7 and Pvm17 departures were found at three sites. Micro‐Checker analysis found null alleles loci in only four cases: in Chinchorro (Pvm32), Xcalak (Pvm46), Utila (Pvm11), and Venezuela (Pvm12). However, both Hardy–Weinberg equilibrium departures and null alleles resulted from heterozygotes excess. Micro‐Checker data analysis pooling all individuals in one group found only one null allele (Pvm15). Nonetheless, it was a false positive because some of the organisms had an interallele at this Locus (204 bp).

**Table 1:** Summary of the microsatellites results used in *Pterois volitans* samples from the Gulf of Mexico and the Caribbean

Locus	SR	Repeat	*N*	Na	Ne	Ho	He	*F*_IS_	*F*_ST_
Pvm4	239–287	(AGAT)12	470	13	9.289	**0.955**	0.892	−0.118	0.041
Pvm7	256–304	(AGAT)9	467	13	6.887	**0.846**	0.855	−0.032	0.040
Pvm11	188–224	(GGAT)9	471	10	3.906	**0.862**	0.744	−0.230	0.060
Pvm12	181–241	(ACAG)11	459	14	6.940	**0.867**	0.856	−0.008	0.017
Pvm15	178–226	(ATCC)7	462	14	5.320	**0.758**	0.812	0.006	0.063
Pvm17	213–261	(GATT)9	472	13	4.766	**0.869**	0.790	−0.171	0.085
Pvm21	165–249	(AGAT)11	474	20	3.765	0.772	0.734	−0.122	0.116
Pvm31	146–194	(ACT)9	469	16	7.859	**0.908**	0.873	−0.134	0.085
Pvm32	175–199	(ATC)10	467	8	2.936	**0.687**	0.659	−0.235	0.146
Pvm37	219–234	(AAT)9	467	6	3.286	0.717	0.696	−0.045	0.049
Pvm42	204–225	(ATC)11	472	8	3.148	**0.833**	0.682	−0.280	0.044
Pvm46	211–246	(GACTT)9	467	8	3.948	**0.812**	0.747	−0.159	0.060

Note

Significant departures from Hardy–Weinberg equilibrium are in bold.

Abbreviations: *F*_IS_, Coefficient of inbreeding; *F*_ST_, Genetic differentiation; He, expected heterozygosity; Ho, observed heterozygosity; Na, number of alleles; Ne, number of effective alleles.

All loci had negative inbreeding coefficient (*F*_IS_) values, which indicated that individuals were less related than expected under a random mating model. The total number of alleles, average alleles, and number of private alleles were higher in the Mexican Caribbean sites (Table 2). A total of 26 private alleles were distributed across six sites (Table 3). Locus Pvm32 had two noticeable private alleles with high frequencies; in the site of Utila, allele 178pb had a frequency of 26.0%, and at Cozumel, allele 196pb presented a 9.7% frequency.

**Table 2:** Genetic diversity for 12 microsatellites loci of *Pterois volitans* by MPA

MPA	*N*	Mean *N* alleles	Total *N* alleles	*N* private alleles	Ho	He	*F*_IS_
TUX	38	6.500	78	0	0.836	0.738	−0.151*
VER	13	6.000	72	1	0.849	0.715	−0.207*
COZ	76	8.500	102	6	0.860	0.754	−0.148*
XKL	89	8.750	105	8	0.787	0.754	−0.051*
CHI	77	8.500	102	5	0.862	0.753	−0.137*
GUA	43	6.667	80	2	0.857	0.726	−0.187*
UTI	54	7.500	90	4	0.783	0.720	−0.092*
ROQ	32	6.083	73	0	0.708	0.692	0.004
VEN	55	7.250	87	0	0.845	0.734	−0.164*

Abbreviations: *F*_IS_, fixation index; He, expected heterozygosity; Ho, observed heterozygosity; *N*, sample number.

* Statistical significance.

**Table 3:** List of private alleles for each *Pterois volitans* collection MPA

MPA	*S*	Locus	Allele	Freq
VER	13	Pvm21	249	0.038
COZ	75	Pvm7	296	0.020
	76	Pvm12	221	0.013
	76	Pvm21	197	0.053
	72	Pvm32	190	0.021
	**72**	**Pvm32**	**196**	**0.097**
	72	Pvm32	199	0.042
XKL	84	Pvm15	182	0.024
	84	Pvm15	190	0.012
	89	Pvm17	261	0.006
	89	Pvm21	201	0.006
	89	Pvm21	209	0.017
	89	Pvm21	213	0.028
	89	Pvm21	221	0.006
	89	Pvm42	225	0.006
CHI	76	Pvm4	287	0.013
	75	Pvm15	222	0.013
	75	Pvm15	226	0.013
	77	Pvm31	161	0.006
	77	Pvm31	194	0.006
GUA	44	Pvm31	167	0.023
	43	Pvm46	211	0.012
UTI	53	Pvm11	216	0.028
	53	Pvm11	224	0.019
	**53**	**Pvm32**	**178**	**0.264**
	54	Pvm42	222	0.009

Note

The values marked in bold match the Pvm32 with the highest frequency.

Abbreviation: *S*, Sample number.

**Figure 2:**
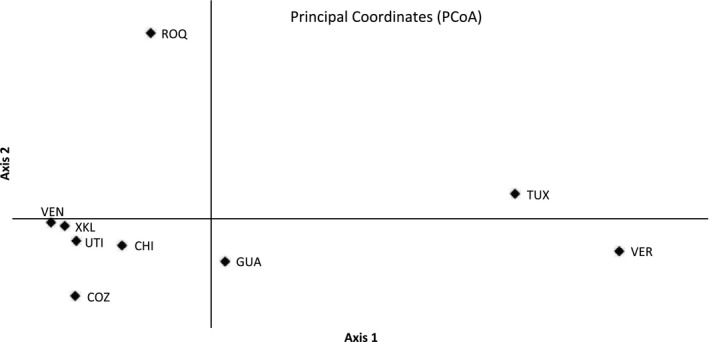
Principal coordinates analysis (PCoA) plot based on pairwise *F*_ST_ index of 475 multi locus genotypes of *Pterois volitans* computed among localities. Tuxpan (TUX), Veracruz (VER), Cozumel (COZ), Chinchorro (CHI), Xcalak (XKL), Guatemala (GUA), Utila (UTI), Chiriviche (VEN), and Los Roques (ROQ)

The first axes of the PCoA explained 43.7% and 18.49% of the variation of the allelic configuration. The first axis of the PCoA shows two clear clusters (Figure 2); one from the Venezuela, Xcalak, Utila, Chinchorro, and Cozumel MPAs; and a second from the Tuxpan and Veracruz MPAs. In addition, the Guatemala and Los Roques sites' data were found isolated from the two main clusters described.

We found small to moderate genetic difference across sites (Table 4). The lowest genetic differentiation occurred between Venezuela and Xcalak (*F*_ST_ = 0,012). The highest differentiation values were found between Los Roques and Veracruz MPAs (*F*_ST_ = 0,074). All genetic differences between sites were significant, even after a sequential Bonferroni adjustment for multiple tests. Additionaly we obtained pairwise Hedrick's standardized $F_{\mathrm{ST}}^{\prime }$ index (Table 5), for all interested in will make comparison between studies with different types of markers and different population sizes. For more information about of the distribution of F_ST_ data, please go to supporting information.

**Table 4:** Multilocus estimates for pairwise *F*_ST_ (below) and *F*_ST_ *p* values (above) at 12 microsatellite loci in the nine sampled MPAs

	TUX	VER	COZ	XKL	CHI	GUA	UTI	ROQ	VEN
TUX		**0.002**	**0.001**	**0.001**	**0.001**	**0.001**	**0.001**	**0.001**	**0.001**
VER	0.022		**0.001**	**0.001**	**0.001**	**0.001**	**0.001**	**0.001**	**0.001**
COZ	0.048	0.067		**0.001**	**0.001**	**0.001**	**0.001**	**0.001**	**0.001**
XKL	0.043	0.063	0.025		**0.001**	**0.001**	**0.001**	**0.001**	**0.001**
CHI	0.043	0.052	0.018	0.014		**0.001**	**0.001**	**0.001**	**0.001**
GUA	0.035	0.053	0.033	0.021	0.028		**0.001**	**0.001**	**0.001**
UTI	0.046	0.067	0.031	0.018	0.025	0.036		**0.001**	**0.001**
ROQ	0.047	0.074	0.049	0.034	0.035	0.047	0.045		**0.001**
VEN	0.045	0.066	0.020	0.012	0.020	0.026	0.020	0.034	

Note

All *F*_ST_ *p* values are in bold because all are significant.

**Table 5:** Multilocus estimates for pairwise Hedrick's standardized $F_{\mathrm{ST}}^{\prime }$ (below) at 12 microsatellite loci in the nine sampled MPAs

	TUX	VER	COZ	XKL	CHI	GUA	UTI	ROQ	VEN
TUX	0.000								
VER	0.080	0.000							
COZ	0.334	0.427	0.000						
XKL	0.303	0.392	0.176	0.000					
CHI	0.298	0.322	0.122	0.093	0.000				
GUA	0.221	0.302	0.218	0.130	0.180	0.000			
UTI	0.302	0.402	0.208	0.114	0.159	0.226	0.000		
ROQ	0.276	0.419	0.305	0.204	0.214	0.275	0.262	0.000	
VEN	0.299	0.407	0.134	0.072	0.130	0.160	0.122	0.197	0.000

**Figure 3:**
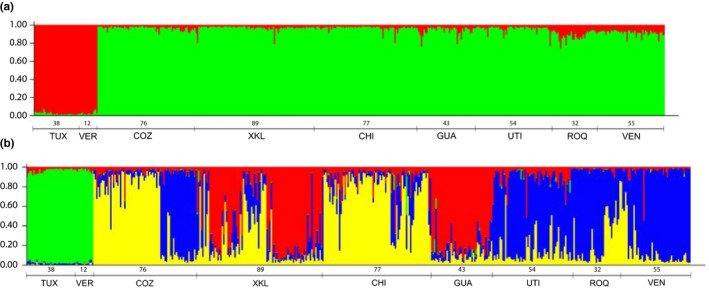
Bayesian clustering of 475 multi locus genotypes of *Pterois volitans*. Each individual is represented by a thin vertical line, which is divided by colored segments representing the percentage of each estimated *K* association. The lines below the graph show the sampling MPAs and the number of individuals

**Figure 4:**
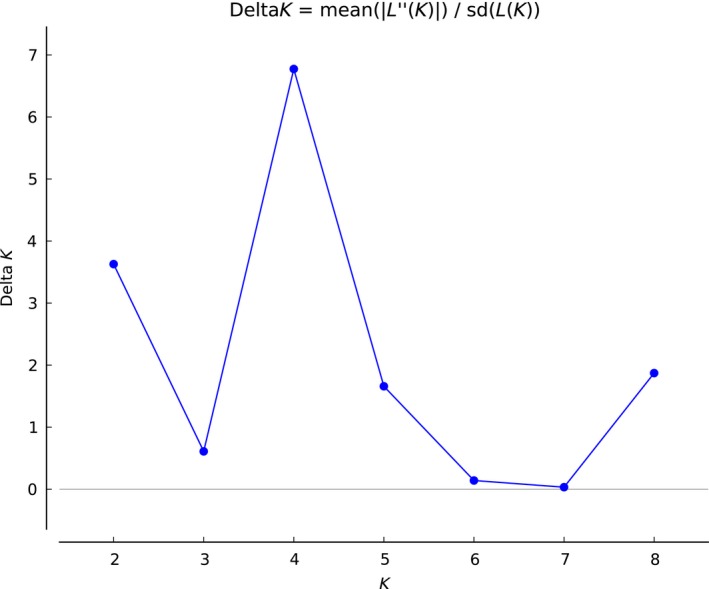
Modeling of number of clusters in *Pterois volitans* using STRUCTURE. ∆*K*, calculated according to Evanno et al. (2005), plotted against the number of modeled genepools (*K*)

The number of genetic groups in the Bayesian analyses computed with all samples suggests that the best subdivision is found with ∆*K* = 4, followed by ∆*K* = 2 (Figure 3a,b). ∆*K* = 4 was also the model that best fit with the Evanno et al. (2005) method (Figure 4).

Using a ∆*K* = 2 shows the organisms captured at the Gulf of Mexico clustered differently than the Caribbean. This difference is also evident in ∆*K* = 4 (Figure 3). The predominant group (34.5% of individuals) is distributed throughout the Utila, Los Roques, and Venezuela populations (blue group); the next group (29%) is present in Chinchorro and Cozumel (yellow group). The third group (22%) is located at Guatemala and Xcalak. The Gulf of Mexico (green group) is a compact group encompassing 10% of the samples (Figure 3).

## 4 DISCUSSION

### 4.1 Genetic structure and connectivity on the regional and local scales

Present results provide evidence of local genetic differentiation among the *P. volitans* along the Mexican coasts from the Gulf of Mexico to the Caribbean Sea. Such local genetic heterogeneity in a species that recently invaded the area raises questions about the routes taken by the invaders. Finding restricted connectivity elucidate the prevailing theories of how *P. volitans* spread along the tropical Atlantic coast, considering that this species has biological characteristics that promote survival success, such as massive reproduction (Morris et al., 2008, 2011), long pelagic larval duration (Ahrenholz & Morris, 2010), tolerance of physical and chemical factors variations (salinity and depth; Jud et al., 2014), and broad trophic diversity (Valdez‐Moreno et al., 2012). Based on the above, it would be expected that the distribution of the allelic configuration of *P. volitans* in the tropical Atlantic would show tendencies toward homogeneity. However, our results demonstrated, instead, an important structure pattern in the studied sites. It should be noted that in some previous studies they have obtained results that were also interpreted to imply restricted connectivity between the Gulf of Mexico and the Caribbean (Betancur‐R et al., 2011; Johnson et al., 2016). The work of Betancur‐R et al. (2011) mentioned that of the nine haplotypes that were in the Bahamas, only four were found in the south of the Caribbean. Johnson et al. (2016) found slight gene restrictions between the Caribbean and the Gulf of Mexico. Our results offer a view of the same patterns with a finer resolution. The interesting thing is the structure of the populations was preserved in some nearby areas, such as Cozumel (Figure 3, *K* = 4). The distribution of *P. volitans* genetic frequencies of our study, suggests strong self‐recruitment and that the main dispersal factor, may be the influence of mesoscale phenomena, transporting larvae from one site to another (Johnston & Purkis, 2015).

When considering this hypothesis, the question is then how a species that is able to colonize rapidly the entire American Atlantic coasts will remain isolated after an initial colonization and not receive new migrants from elsewhere? At this stage, we can only formulate hypotheses and consider just the initial colonization. If we consider that the offspring from the initial colonization will rapidly occupy the suitable habitat, then new migrants will face intraspecific competition on a species that shows strong cannibalism (Dahl et al., 2018; Valdez‐Moreno et al., 2012). In the end, the original colonizing genotype(s) may remain locally dominant. Based on this process, we can then find in the end local heterogeneity in genetic pools, with a remaining signal of migration driving connectivity patterns. The presence of private alleles with high frequency in Cozumel, Chinchorro, and Xcalak MPAs suggests such an immigration process as well with the arrival of single unique genetic pools.

The genetic structure can be linked in some way to the local Mesoamerican Reef System currents that coincide with a mesoscale temporal gyre associated with the meandering of the Cayman Current that can connect between south Cuba and the West Yucatan peninsula (Carrillo, Johns, Smith, Lamkin, & Largier, 2015; Huang, Walker, Hsueh, Chao, & Leben, 2013). These currents could be transporting larvae from Southeastern Cuba to the West Yucatan and keep isolated reefs as those from Xcalak and Guatemala. The Gulf of Honduras also acts as a geographic barrier promoting a pattern of counterclockwise circular currents that keeps larvae near the coast and restricts migration to adjacent areas. Our results agree with the patterns of the currents of the Mesoamerican Reef System hydrography and with the flow pattern's cyclonic circulation known as the Honduras Gyre (Carrillo et al., 2015). Still, the finding of *P. miles* in Banco Chinchorro (Guzmán‐Méndez, Rivera‐Madrid, Díaz‐Jaimes, García‐Rivas, et al., 2017) evidence that the dispersion process of both species (*P. miles* and *P. volitans*) is constant and increasing. It is likely that at some point all haplotypes will be dispersed throughout the entire Caribbean Sea.

The success of the MPAs depends on the dispersion of species being greater than the distance between protected areas (Beltrán, Schizas, Appeldoorn, & Prada, 2017). This criterion is only partially fulfilled among some marine areas that we considered, for example, Chinchorro‐Cozumel and Xcalak‐Guatemala (both with very similar physiographic characteristics). Utila, Chiriviche, and Los Roques have greater connectivity even when there is a greater geographical distance. The information that we present is a useful connectivity indicator for MPAs because the behavior of this population of invasive fish could reflect restricted connectivity of other noninvasive species.

Since the beginning of analyses based on the mitochondrial control region (mtDNA D‐loop), it was mentioned of possible slight restrictions on the genetic flow of *P. volitans* between the Caribbean and the Gulf of Mexico (Betancur‐R et al., 2011; Butterfield et al., 2015; Johnson et al., 2016; Toledo‐Hernández et al., 2014; Freshwater et al., 2009). The study of Johnson et al. (2016) conclude that there is no detectable direct flow in the Gulf of Mexico from the North Atlantic and that the invasion of Gulf of Mexico probably was originated by migrants from the Caribbean Sea. Although mitochondrial analysis has offered valuable information on the dynamic of this species, present microsatellites data are providing a new perspective. Our analysis indicates that the samples from the MPAs in the Gulf of Mexico and the Caribbean Sea are characterized by four different genetic groups with a high probability of belonging to the group. We observe that individuals from the Gulf of Mexico form a group with a distinct configuration that differs from the genetic structure of the groups from the Caribbean Sea (Figure 3a,b). This could be the result of a bottleneck, caused by a minimum number of organisms being able to arrive and proliferate in the Gulf of Mexico. The current results also show a private allele in the samples from the Gulf of Mexico, which probably comes from a population different from the population collected in the Caribbean. The presence of an interallele (Pvm15: 204) distributed in most locations—with the exceptions of Cozumel and Guatemala—reminds us that although the Gulf of Mexico and the Caribbean Sea are identified as different genetic groups, these groups share 74% of their alleles.

The invasion of the Gulf of Mexico has been slower than that of the Caribbean, its genetic group differs and has a unique allele not identified in Caribbean samples. Additionally, there is only 63% of the allelic similarity between samples from the Gulf of Mexico and those from Cozumel—the geographically closest collection MPA. Based on these facts and the results of the recent study by Pérez‐Portela et al. (2018), we hypothesize that this group comes from a different invasion route. It is probable that the group collected in the MPAs of the Gulf of Mexico has invaded slowly because it has bordered counterclockwise off the coast of Florida and Gulf of Mexico, and has followed a path from east to west, traveling countercurrent.

### 4.2 Fine scale genetic differentiation

The genetic structure we found shows a particular pattern regarding the distribution of the Cozumel samples. When we considered four genetic units, the Cozumel samples get divided in two units: 48 individuals belonging to one genetic group and another unit of 28 individuals. This difference is quite pronounced and clearly due to the sampling process that involved a collection of 48 individuals at a single time and in a unique location, while the other 28 were collected at a different time, on the same day, but about 3 km north of the first collection site, at the borderline of the Marine Protected Area, adjacent to the area where local fishermen constantly catch *P. volitans* to sell. Considering that *P. volitans* is a territorial organism (Jud & Layman, 2012), we believe that we are finding evidence of a different cohort settlement in the north of the Cozumel MPA, originating from limited reproduction events (Johnson et al., 2016). This outcome provides a new perspective on the functioning of the lionfish colonization which suggests that recruitment happens in a wave of related individuals issued from single reproductive events. It is important to note that as part of the management of their issues with reef areas, all the countries that were affected by the invasion of *P. volitans* in the Western Atlantic have generated campaigns to remove them. Our results observed in Cozumel (Figure 3, *K* = 4) may be showing that the intensive removal of organisms has an effect on settled populations. In this light, we might expect that, depending on the intensity of the removal of organisms, some areas with greater dynamics of migrants will have a fluctuating variability of genetic groups derived from the settlement of new migrants.

### 4.3 Could differences in the type of reefs cause settlement differentiation?

It is worth mentioning that the Marine Protected Areas where the collections were made differ in the type of reefs they contain, and those that have some similar physical characteristics also show a similarity in the genetic group identified in Figure 3 (*K* = 4). The Gulf of Mexico reefs present characteristics totally different from those of the Caribbean, due to a greater introduction of fresh water, sediments from rivers, estuaries, and lagoons that flow into its waters. It is predominantly rocky with low coral cover, poor visibility, and relatively shallow waters (González‐Gándara, 2015). By contrast, the Caribbean reefs present oligotrophic waters, with little contribution of fresh water, low continental sediments, and warmer temperatures, as well as a wider depth range. And each site has specific characteristics, Cozumel and Chinchorro—relatively distant from the Yucatán Peninsula—encircle islands, (Jordán‐Dahlgren & Rodríguez‐Martínez, 2003). Xcalak and Guatemala present barrier reefs, parallel to the coast, but they have been affected by anthropogenic activities (Almada‐Villela, Mcfield, Kramer, Kramer, & Arias‐Gonzalez, 2002). The reefs of Utila Honduras present shallow platforms and walls with free fall of the continental slope (Weil, 2003). Los Roques is a reef type atoll, which is located far from the coast of Venezuela (Weil, 2003). Chiriviche in Venezuela is dominated by mangrove forests that line the coast and includes reefs around small calcareous islands patches (Weil, 2003). This is interesting because it suggests that the genetic differentiation of this invasive species could be influenced by the natural selection. This should be further analyzed with more specific studies that could lead to a complementary interpretation illuminating more thoroughly the distribution of this species.

### 4.4 The probable number of the founding population

The first hypothesis about the spread of *P. volitans* suggests that the invasion began with six to eight individuals that escaped from an aquarium in Miami, Florida, USA (Courtenay, 1995; Morris & Akins, 2009; Whitfield et al., 2002). Betancur‐R et al. (2011) mentioned the minimum number of founding individuals required to explain the observed genetic diversity in WA lionfish it was between eight and 12. Our work revealed that the number of alleles at Pmv21 Locus was 20 (Table 1), suggesting that the number of original invasive organisms must have been at least more than 10, based on the conservative assumption that each organism had a unique pair of alleles, and without taking into account the probability of allele mutations. A recent estimate suggests a minimal source population of 118, and when accounting for allele effects, the number of colonizers increased to 272 (Selwyn et al., 2017). The conclusion of Selwyn and collaborators is more consistent with the probability of occurrence of the results that we obtained in our study.

Present microsatellites data show HW disequilibrium for excess of heterozygotes. Taking into account the study of Selwyn et al. (2017) and comparing the first studies that mentioned that the number of mitochondrial DNA Haplotypes of *P. volitans* is nine (Freshwater et al., 2009), it could be considered that some founding organisms were probably related or belonged to the same populations.

The excess of heterozygotes found in the study could be attributed to biological realities of this *P. volitans* population, because it violates the criteria of an ideal population (Selkoe & Toonen, 2006).

Although it is known that populations that present a recent bottleneck show apparent excess of heterozygotes (Cornuet & Luikart, 1996; Sakai et al., 2001), our analyses found heterozygosis in all loci; therefore, it is likely to be a reflection of the founder effect noted in other studies (Freshwater et al., 2009). Even so, this information differs with the studies of Pérez‐Portela et al. (2018) and Bors et al. (2019). However, the differences may be inherent to the different molecular markers used.

Overall, our data clearly show that the early invasive history of the lionfish in the Atlantic is not as simple as a few individuals released around the 1980s. Also, this species is extremely faithful to its territory and that its movement as an adult is relatively limited to a few hundred meters (Jud & Layman, 2012). In this context, it is more likely this invasion was caused by the introduction of more than a hundred organisms of *P. volitans* from the same geographical area, and that were released in a single exhibition, that diverse introductions of organisms that probably took place at different chronologic moments within the last 30 years. Which makes us think about the probability that the black market of ornamental marine fish was behind of the cause of the massive liberation in an entrance port. Which of these hypotheses is close to reality? It is material for another study. But the history of the stepwise invasion in the Atlantic should be revisited in light of this approach.

## 5 CONCLUSION

The origin of the Atlantic Lionfish invasion, the number of individuals that initiated it, and the connectivity patterns in the Caribbean and the Gulf of Mexico is more complex than has been previously suspected. Our findings demonstrate that there is genetic differentiation on both regional, local, and fines scales. We suggest that the colonization of each geographic area has been in temporary pulses, probably caused by mesoscale phenomena, and intensified by self‐recruitment. The presence of private alleles in the Mexican Caribbean points to the need to carry out further studies in that area, since it presents greater dispersal dynamics of larvae. These results open new challenges in research seeking to understand the functioning, the evaluation, the efficiency, and the management strategies of MPAs in the Caribbean and the Gulf of Mexico. Integrating all the components, these results are promising toward controlling this invasive species, acting at local scales that may have real short‐term effects at regional scale.

## CONFLICT OF INTEREST

The authors declare no conflict of interest.

## AUTHORS CONTRIBUTION

Authors whose names appear on the paper have contributed sufficiently to the scientific work and therefore share collective responsibility and accountability for the results. The authors of this manuscript contributed in this project proportionally to the location in which their name is presented in the article. All authors have approved the work, and all authors have agreed to the last version of the manuscript.

### OPEN DATA BADGES

This article has earned an https://openscience.com for making publicly available the digitally‐shareable data necessary to reproduce the reported results. The data is available at https://datadryad.org/stash/share/OF-FgZjZer-iMwDVoglD4C5-Ikr7pIFeoW9Evldhg_s.


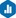


## Data Availability

The data are available from the Dryad https://doi.org/10.5061/dryad.zgmsbcc68
