# Supplementary material for: Genetic connectivity of lionfish (Pterois volitans) in marine protected areas of the Gulf of Mexico and Caribbean Sea
Source: Ecol Evol. 2020 Apr 16;10(9):3844–55. doi: 10.1002/ece3.5829 (PMC7244795; doi:10.1002/ece3.5829)
Supplement: Supplementary file 1 [file ECE3-10-3844-s001.docx]

**Supplementary material**

Fig. 1S. Histogram of the pairwise F_ST_ Multilocus frequencies of alleles in the Gulf of Mexico and the Caribbean.

Fig. 2S. Histogram (by locus) of the F_ST_ Multilocus frequencies of alleles in the Gulf of Mexico and the Caribbean.

Table 1S Allele frequencies of divergence among pops (Net nucleotide distance) of K=4 (Figure 3B), computed using point estimates of P.

|  | 1 | 2 | 3 | 4 |
| --- | --- | --- | --- | --- |
| 1 | - |  |  |  |
| 2 | 0.0242 | - |  |  |
| 3 | 0.0522 | 0.0518 | - |  |
| 4 | 0.0321 | 0.0269 | 0.0656 | - |
|  |  |  |  |  |
